# Influence of Physical Activity and Sport on the Inclusion of People with Visual Impairment: A Systematic Review

**DOI:** 10.3390/ijerph19010443

**Published:** 2021-12-31

**Authors:** Virginia Alcaraz-Rodríguez, Daniel Medina-Rebollo, Antonio Muñoz-Llerena, Jesús Fernández-Gavira

**Affiliations:** 1 Faculty of Education, Valencia International University, 46002 Valencia, Spain; virginia.alcaraz@campusviu.es; 2 Research Group “Social Inclusion, Physical Education and Sport, and European Policies in Research”, University of Seville, 41013 Seville, Spain; amllerena@us.es (A.M.-L.); jesusfgavira@us.es (J.F.-G.); 3 Physical Education and Sport Department, Escuela Universitaria CEU San Pablo, 41013 Seville, Spain; 4 Physical Education and Sport Department, University of Seville, 41013 Seville, Spain

**Keywords:** inclusion, physical activity, sport, visual disability, impairment

## Abstract

People with visual impairment have greater difficulty in accessing physical activity and sport, and a lack of social interaction is also associated with a risk of exclusion. Work is currently being done to include people with visual impairment through physical activity and sport. However, there is a lack of studies examining the status and overall effectiveness of interventions in the pre- and post-COVID stage. This study aimed to provide solid evidence on the characteristics and effectiveness of interventions for the inclusion of people with visual impairment through physical activity and sport in order to address the need for dissemination on this topic. The bibliographic search was carried out with the words “Physical activity”, “Physical exercise”, “Sport”, “Physical training”, “visual disability”, “visual impairment” and “inclusion” in the databases PubMed, Scopus, Web of Science and Google Scholar from 2018 to 2021.The article selection process was according to the PRISMA protocol with a final selection of nine articles. The main results highlighted that the programmes improve the perception of people with disabilities, increase social skills and health and increase the social importance of people with disabilities in the social environment. Among the most generalised conclusions were the need for specialised training, the need for social inclusion and participation of people with visual impairment in their environments and increased physical activity.

## 1. Introduction

The mainstreaming of people with disabilities in general is on the rise thanks to improved policies and public measures. These policies are supported by international standards such as the Charter on the Rights of Persons with Disabilities [1] and the 2030 Agenda [2]. According to WHO reports, in 2015 there were 217 million people with low vision worldwide, of whom 36 million were blind. The WHO estimates a growth between 7% and 10% every five years. In 2020 there will be 277 million people with low vision and 587.6 million people worldwide in 2050. For blind people, the estimates were 38.5 million in 2020 and 114.6 million in 2050 [3].

Visually impaired people suffer from impairment that restricts their perception of their environment and limits, to some extent, their acquisition of psychomotor skills and also, to some extent, their mental, social and physical health [4,5].

Seventy percent of the information our brain processes is related to vision, so visual receptors play a key role in information processing, planning and organisation and recognition of the environment [6]. This is why some people with visual impairment are limited in their access to physical and social development from childhood onwards.

Regardless of the cause of blindness, there is no feedback from the environment, affecting psychological health, personal autonomy, attitude and life in society, with various alterations in the perception of one’s own body in space, alterations in body adjustment and basic motor skills [7], inducing new comorbidities, such as a sedentary lifestyle, that usually lead to obesity or being overweight, adding another risk factor for the cardiovascular/pulmonary system.

Although there are no specific studies on this subject, several authors mention the importance of including blind people in exercise programmes in order to prevent the appearance of new pathologies that may affect their state of health [8].

In addition to this situation, another frequent conditioning factor is the restricted access to sports programmes or targeted exercise for this population group, either due to the lack of dissemination of information or lack of knowledge about architectonic facilitators. The few trained educators and the lack of modified assessment measures to be applied to people with blindness limit the implementation of programmes and the appropriate prescription of activities or exercises [9].

According to the International Classification of Functioning, Disability and Health: disability encompasses impairments, activity limitations and participation restrictions [10], making it a negative term for the sufferer. In the case of visual sensory impairment, its commitment is related to the limitation in functions related to motor qualities such as: balance, stability, postural control and muscle strength, among others, which, ultimately, end up affecting their independence and functionality and limiting their participation in society and performance in their daily activities.

Visual rehabilitation programmes are based on providing comprehensive and timely care in order to facilitate autonomy and functional independence in daily life activities; however, the physical fitness component and the promotion of physical activity are not always addressed.

People with visual impairment encounter barriers to their sport practice such as lack of institutional support (public administration, federations, etc.), personal and family environment limitations, lack of accessibility in the information of sport environments and problems in the accessibility of sport infrastructures [11].

### 1.1. Physical Activity as a Factor for Inclusion

Today, there is no doubt that sport and physical activity, beyond a competitive model, is an essential element in creating environments of tolerance, cooperation, solidarity, health and inclusion. Physical activities and sports offer environments to promote physical, cognitive and value-based learning that may be, in many cases, affected by some kind of disability [12].

Society has historically shown negative attitudes towards people with visual impairment; these attitudes, together with other factors, influence physical inactivity in this group. We have to look at the socio-ecological theory of human behaviour, which focuses on the environmental factors that influence the resulting behavior [12,13]. However, when talking about sport and disability, it is necessary to take into account not only the physical benefits but also the psychosocial benefits. As mentioned above, the practice of physical activities has been shown to be beneficial for the creation of close emotional bonds, the development of social skills and/or social reintegration.

In this article, we look at the documents reviewed for sport and physical activity as facilitators of social inclusion in a sporting practice carried out jointly by people with and without disabilities, adjusting to the possibilities of the participants and maintaining the objective of the sporting speciality in question.

### 1.2. Physical Activity in People with Visual Impairment

Different studies mention that people who are blind show a lower level of physical activity and a greater deterioration of their health than visually impaired people [12,13,14]. The difficulty in this population is mainly related to motor tasks that require strength and speed, typical components of physical activity, causing a higher risk of developing chronic diseases (WHO).

Because adults with blindness are four times more likely to be impaired in performing activities of daily living and five times more likely to have limited mobility than people with adequate visual acuity, regular physical activity has been found to improve functional independence, prevent the risk of falls and improve social relationships, resulting in a better quality of life [14].

The low motivation of people with disabilities to engage in physical activity may be due to a lack of knowledge of its benefits, as well as the constant external blocks derived from those prejudices and barriers imposed by society [15]. For example, Henderson et al. [16], after studying a group of women with physical disabilities, concluded that perceived attitudes, stereotypes and prejudices often limited their participation in sporting activities.

In fact, it can be concluded that the social perception of people with disabilities is the most influential factor in why they do not participate in physical activities [17]. This social perception ends up weighing down the motivation of this group, which many psychologists agree in pointing out as an internal factor that provides energy and directs human behaviour in the field of sport and physical activity and whose study allows us to know why some people choose to do some activities and not others, the factors that are related to this choice and those that determine whether they remain in them or abandon them [17].

Research with blind people is clearly insufficient, especially in the area of physical activity. Specifically, even with the limited published evidence, it has been shown that people with blindness tend to be less physically active than sighted people due to the insecurity of not recognising their environment and being influenced by other socio-demographic factors such as age, gender and type of blindness [14].

In the daily activities of blind people, sedentary behaviours are marked, such as: listening to television programmes, listening to music and activities that do not require greater energy expenditure, etc. At the same time, during these activities, they increase their caloric intake, leading to the aforementioned comorbidities [18].

Although the above are behaviours that are already evident in this population, it is necessary to take into account that blind people have similar physical abilities to sighted people; the big difference is that they show a limitation for learning due to acquired or congenital alterations in the visual system. Therefore, it is necessary to look for other learning strategies based on the experimentation of other information channels, such as the tactile, auditory, proprioceptive, kinaesthetic and affective senses, without leaving aside the motivation towards motor activity due to the fear of the unknown, which can lead to the rejection of the practice of physical activity [19].

There is currently a lack of research that addresses the reality of the inclusion of people with visual impairment in both educational and sporting environments in relation to physical activity and sport.

In this study, we carried out a review study to obtain information on the studies carried out in recent years that address the influence of physical activity and sport on the inclusion of people with visual impairment and, in this way, to some extent, solved the lack of studies in this field and of attention to people with visual impairment.

## 2. Materials and Methods

### 2.1. Search Strategy

The study followed the PRISMA protocol checking list and has been registered in the PROSPERO platform with the registration number 299,414.

This systematic review of the influence of physical activity and sport for the inclusion of people with visual impairment was carried out according to studies published in journals with English as the main or secondary language in PubMed, Scopus, Web of Science and Google Scholar databases. The search keywords were physical activity, physical exercise, physical training or sport, inclusion and visual impairment, with a synthesis of these operators always incorporated in order to find related articles. The article selection process was carried out following the guidelines of the PRISMA protocol [20].

### 2.2. Eligibility Criteria

This scoping review included any empirical, conceptual or peer-reviewed perspective [20]. The selected studies explicitly address the inclusion of people with visual impairment with sport as a mediating factor.

The first search yielded a total of 2539 articles. A temporal filter was applied to this selection of articles, taking only those published between 2018 and 2021.

The eligibility criteria established followed the quality criteria are detailed below:

- Taking into account peer-reviewed publications and those that were final papers, theses, systematic reviews and dissemination documents (341), 2198 articles were left;
- Articles duplicated in the different databases (876) were removed, leaving a total of 1322 articles;
- Those articles in which only the abstract was available (743) were removed, leaving a total of 579 articles;
- Lastly, we applied the filter that at least the abstract should be in English, leaving a total of 251 articles.

During the literature review, two researchers (A.M. and G.F.) reviewed those articles that passed the eligibility criteria and assessed the content of study titles and abstracts and their relation to the research objective of the present work.

After this last part, a total of 9 articles meeting all the eligibility criteria presented were reviewed and fully analysed.

### 2.3. Data Analysis

The text data from the primary studies were analysed using conventional content analysis. After repeated evaluation of the results, the primary codes were recognised and classified into different groupings according to the degree of similarity.

### 2.4. Synthesis of Results

In this synthesis, an interpretative quadrant was developed in which the primary and central themes addressed were included. In order to carry out this qualitative synthesis, the review texts available in full were analysed. In this case, all the data and conclusions of the articles were analysed qualitatively and as a whole, showing that the same themes and content were addressed. Specifically, quantitative data were not numerically aggregated with data from other quantitative articles because they were not relevant to the research objective itself.

Finally, to help the reader discern whether the reported material came from empirical or perspective articles, the synthesis explicitly reported some study characteristics, populations or any numerical findings where appropriate. Finally, as described in the study protocol, we conducted a last stage of consultation with a preliminary version of the results and their discussion.

## 3. Results

**Figure 1:**
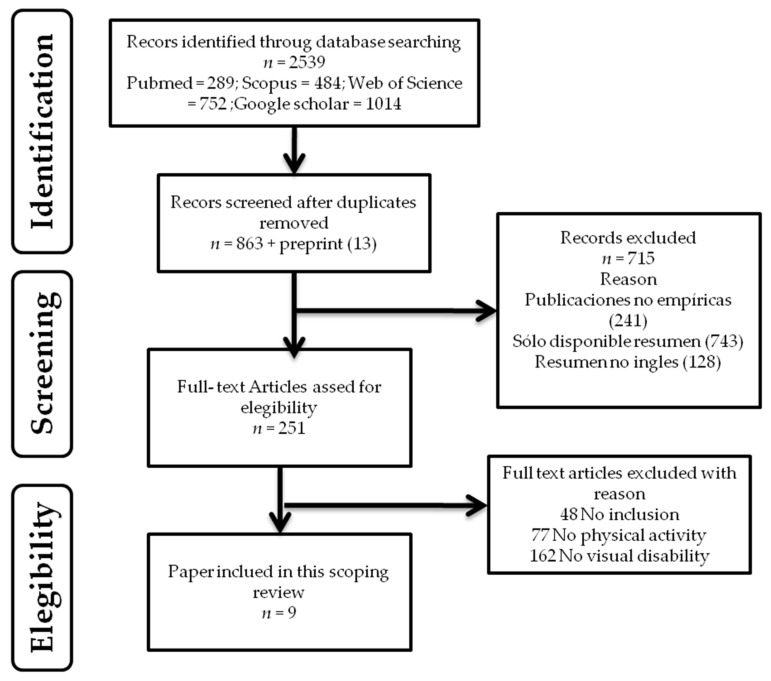
PRISMA flowchart of the scoping review with thematic analysis.

Figure 1 provides the flowchart of this review and how it transitioned from 2539 initial references to thenine articles included in the final analysis.

Table 1 presents the descriptive register of the articles, indicating as basic data the title of the study, author and year, type, specific group and results.

Table 2 shows the articles that were finally analysed and how they were distributed by type of publication and source and by the conditions of inclusion.

### 3.1. Thematic Analysis: General Description

The thematic analysis of the reviewed literature unravelled different types of benefits and effects of physical activity and sport programmes on social participation and inclusion in the proposal’s own and close environment.

Some of the main themes addressed were participation in physical activity as a factor in increasing physical health and increasing social competences through sport. A recurring theme was the problem of accessibility and access to leisure-time sport activity.

Finally, all these themes stemmed from these central issues: the lack of disability-inclusive responses and preparedness for the care of people with disabilities, the disadvantages of physical activity before and during the pandemic, emergencies and the disadvantages experienced by people with visual impairment.

### 3.2. Primary Themes

The primary themes addressed in the articles analysed were school physical activity as a factor of inclusion, specialisation of professionals as a way of inclusion, sport programmes of clubs and associations, improvement of health through inclusion and improvement of psychosocial factors through physical sport practice.

#### 3.2.1. School Physical Activity as a Factor of Inclusion

Physical education for inclusion does not only aim at incorporation into the system, but also at incorporation into socialisation and integral education. It is not only educational inclusion but inclusive education.

Educational centres and professionals are responsible for exclusion within the classroom and sports activities. The context is constantly changing, and the perception is that the school does not change. Teachers and professionals do not have the resources to respond to these changes and, therefore, there is a perception of a lack of empowerment on the part of learners [26].

Students with visual educational barriers do not have the tools that allow them to be part of the class and are excluded in a certain way by their classmates and even by the teacher himself. It is concluded that it is the lack of knowledge on the subject that is the cause of this reality [30].

Pupils and teachers alike lack awareness and knowledge about inclusion and the characteristics of people with disabilities. When awareness is raised, everything becomes easier and more sensible [21,31,32].

#### 3.2.2. Inclusion in Physical Activity as an Element of Barrier Removal

Activity in daily life is one of the main aspects of a healthy life. Therefore, barriers which prevent children and young people with disabilities from being more active need to be reduced so that they can also benefit from the physical and social advantages [21,22,27,28].

Most children and young people with visual impairment want to do more physical activity and sport but lack motivation due to the lack of accessibility [11,29].

It is, therefore, a priority to provide the necessary support to clubs, sports centres and families to meet this need [5,22].

Sports clubs should expand their programmes and make them more attractive for children and young people with disabilities. At the same time, information about the need for appropriate programmes is needed to reach parents and their children, e.g., by stimulating cooperation between special schools and sports clubs [21,22].

Furthermore, professional coaches should increase their efforts to inform parents about the strengths and positive effects of sports and decrease their concerns about their child’s participation in organised sports [11,22,33].

#### 3.2.3. Sports Programmes of Clubs and Associations

Most people with visual impairment feel excluded on some occasion during their sport and physical activity [12,25]. Children and young people in educational institutions have an additional factor of helplessness compared to adults because of the possible rejection within the institutions [15,23].

For inclusion to be real, participants with visual impairment within clubs must be autonomous and have control over the support they receive or need to receive [32,34].

Autonomy-supportive and mastery-oriented climates for participants and enabling a higher level of enjoyment increase motivation to engage in sport [28,32,33,34].

If conditions do not promote diversity awareness or mastery experiences within a heterogeneous group of people, those with visual impairment or other disabilities may feel excluded [23].

If we understand disability as the result of the interaction between individual attributes and contextual conditions, as in the interactional approach to disability, it becomes important to explore how we can alter the way physical activity and sport is constructed in response to needs, rather than excluding those with differences [28,29,32].

#### 3.2.4. Improving Inclusion with Trained Professionals

Inclusive physical activity, physical education and sport rely heavily on the training of professionals [11].

In the case of the analysed articles, the focus is on the opinion of students with disabilities and how they perceive the attention of their teachers and coaches in sport and physical activities [25].

Students with visual impairment consider that their inclusion in sport and physical activities is not deficient but could be improved with the preparation of their teachers [11,25,34].

The curricular adaptations made by the teacher are fundamental for the students to be able to participate without exclusion, and they also think that educational establishments do not provide the material resources and support for inclusion in this subject, which are key elements to carry it out [32,34].

The participants with visual impairment feel that they are accepted and included in the educational activities by small groups of peers and not as a whole, so that the processes of sensitisation of the whole educational community in general are relevant aspects to take into account in order to favour the inclusion of all students [25,32].

For people with visual impairment, participation in sporting activities is very important, particularly for health, aesthetics and fun; they are clear about the importance and benefits of physical activity for the integral development of the human being [25,27].

Although it is true that, in recent times, there have been changes in the subject of inclusion, these have been gradual, and there are still many aspects to be resolved, especially the barriers that hinder educational inclusion, such as lack of professional development, lack of access to appropriate materials and lack of awareness, among others.

#### 3.2.5. Improving Psychosocial Factors through Physical Exercise

The lack of accessibility in facilities, activities and physical activity and sports programmes makes visually impaired people feel less accepted and adapted for because they cannot perform like the rest of the participants, increasing this feeling of rejection in people with multiple disabilities.

Visually impaired people value as an inclusion factor that the people who attend them as technicians and those who share the activity have an attitude and an interest in the knowledge of people with disabilities. Another factor is that the professionals have knowledge and control over the physical activity carried out. To these are added the personal predisposition as a facilitating element for social success and the elimination of exclusion factors [24,26,29].These properties are coupled with the needs for physical competence, personal autonomy and personal affinity (kinship or friendship), as presented in self-determination theory [27,28,29,35].

People with visual impairment will be more motivated if, within the activities, they feel they are included and there are positive personal interactions that satisfy their personal needs for recognition of competence, autonomy and personal relationships, which are fundamental elements for social inclusion [28,30,32,34].

#### 3.2.6. Improving Health through Inclusion

The health of people with disabilities is worsened when they do not have an adequate and optimal practice of physical activity and sport, and increased accessibility improves the health of people with visual impairment [5,27,28,32].

These results should lead both public and private institutions to develop initiatives for the promotion of the health of people with disabilities.

## 4. Discussion

Physical education for inclusion does not only seek incorporation into the system, but also incorporation into socialisation and integral training, which is why we speak not only of educational inclusion but also of inclusive education.

Educational centres and professionals are responsible for exclusion in classrooms and sports activities because they do not attend to the characteristics and specific needs of students with visual impairment [31,32]. The context of the school and society are constantly changing, but the perception of students with visual impairment is that its attention does not change, and teachers do not have the resources to respond to these changes and, therefore, there is a perception of a lack of awareness and a lack of training on the part of students [31].

Students with visual educational barriers do not have the tools that allow them to be part of the class, being excluded in a certain way by their classmates and even by the teacher him/herself due to lack of knowledge of the situation causing the exclusion [32].

Students without disabilities, as well as specialist teachers, lack awareness and knowledge about inclusion and the characteristics of people with disabilities but, with sufficient training, everything is easier and simpler [21,32].

Activity in daily life is one of the main aspects of a healthy life, and, in order for people with visual impairment to benefit from these physical and social aspects, barriers must be removed or reduced, and the possibility for them to be more active must be given [11,22,27,28,29,33].

Visually impaired people want to do more physical activity and sport in their leisure time but lack motivation due to the lack of accessibility on both the institutional and social level [11,28,33]. It is, therefore, a priority to provide the necessary support to clubs, sports centres and families to meet this need [5,22].

Sports clubs should expand their offer of physical activities to make them more attractive to all people with visual impairment. In addition, coordination between entities, such as special education centres, disability care entities and others, is necessary [21,22].

In addition, professional coaches should increase their efforts to inform parents about the strengths and positive effects of sports and decrease their concerns about their child’s participation in organised sports [11,22,34].

Most people with visual impairment feel excluded at some point during their physical activity in sport [11,23]. Children and young people in schools have an additional factor of helplessness compared to adults because of the possible rejection within schools [15,23].

For inclusion to be real, participants with visual impairment within clubs must be autonomous and have control over the support they receive or need to receive [32,33].

An autonomy-supportive and mastery-oriented environment for participants and enabling a higher level of enjoyment increase motivation to engage in sport [28,29,32].

If conditions do not promote diversity awareness or mastery experiences within a heterogeneous group of people, those with visual impairment or other disabilities may feel excluded [23].

If we understand disability as the result of the interaction between individual attributes and contextual conditions, as in the interactional approach to disability, it becomes important to explore how we can alter the way physical activity and sport areconstructed in response to needs, rather than excluding those with differences [32].

Inclusive physical activity, physical education and sport rely heavily on the training of professionals [11].

In the case of the analysed articles, the focus is on the opinion of students with disabilities and how they perceive the attention of their teachers and coaches in sport and physical activities [25].

Students with visual impairment consider that their inclusion in sport and physical activities is not deficient but could be improved with the preparation of their teachers [11,24,34].

The curricular adaptations made by the teacher are fundamental for students to be able to participate without exclusion, and they also think that educational establishments do not provide the material resources and support for inclusion in this subject, which are key elements for carrying it out [32,34].

People with disabilities feel that they are accepted and included in sporting activities by small groups of peers and not as a whole, so awareness-raising processes for the entire educational community in general are important aspects to take into account in order to favour the inclusion of all students [25,32].

For participants with visual impairment, participation in sporting activities is very important, particularly for health, aesthetics and fun; they are clear about the importance and benefits of physical activity for the integral development of the human being [25,31].

Although it is true that, in recent times, there have been changes in the area of inclusion, these have been gradual, and there are still many aspects to be resolved, especially the barriers that hinder educational inclusion, such as lack of professional development, lack of access to appropriate materials and lack of awareness, among others.

The lack of accessibility in facilities, activities and physical activity and sports programmes makes visually impaired people feel less accepted and adapted for because they cannot function like the rest of the participants. In addition, people with multiple disabilities are also affected by negative social treatment.

A nivel social todas las personas destacan como factores de inclusion que las personas se presenten, el interes por el concimiento, tener el control de la situación, la posibilidad del exito social y eliminación de los elementos de exclusion [26,32,34]. Estos atributos se alinean con las necesidades de competencia, autonomía y parentesco como se articula en la teoría de la autodeterminación, una teoría de la motivación.

Los participantes estarán motivados y se sentirán incluidos si las actividades e interacciones satisfacen sus necesidades de competencia, autonomía y afinidad. La accesibilidades vital para la inclusion social [26,32,34].

Las personas con discapacidad suelen tener una salud peor que las personas sin discapacidad en las mismas condiciones por la falta de acceso a la actividad física y la falta de motivación en la práctica, todo resultante de la falta de actividad física [5,27,28,31,32].

Las instituciones tanto públicas como privadas deben desarrollar iniciativas para la promoción de la salud de las personas con discapacidad.

## 5. Conclusions

People with a visual impairment have a lower level of physical activity and sport than people without a disability. This situation is aggravated when the person is blind, further reducing the level of physical activity and social interaction through sport and physical exercise.

Visual impairment should not limit people’s physical activity as long as there are social policies that enable people to have optimal access to sport in school, leisure time and performance. Blindness does not have to limit the practice of physical activity, but, on the contrary, it is necessary to consider the possible barriers that restrict these people from participating in physical activities.

In this sense, only when the person with blindness perceives that he/she can perform new physical exercises autonomously, without the need of a sports guide, can he/she have a greater perception of competence and autonomy in the sports field.

Sports activities have positive relationships with all domains of quality of life in people with visual impairment. Therefore, appropriate sport activities will increase the success of rehabilitation programmes and their quality of life.

The COVID-19 pandemic has resulted in billions of people being placed in conditions of social distance, isolation or quarantine worldwide. Compliance with these measures brings with it public health problems related to decreased physical activity, increased sedentary lifestyles and a psychological impact associated with the state of uncertainty in the case of people with visual impairment and the possibility of physical activity was reduced; however, the weakness of this study is that no document has been produced that addresses these limitations in this population.

This study was proposed to solve, to some extent, the lack of studies on physical activity and sport in people with visual impairment and its importance in contributing to an improvement in the quality of life and both physical and psychosocial health. For this reason, we believe it is important to continue researching this subject and to be able to advance and improve the social inclusion of people with visual impairment through physical activity and sport.

The aim of this review was to discover, analyse and recommend measures on physical activity and sport to promote the social inclusion of people with visual impairment through the analysis of documents. Evidence was found that physical activity and sport proposals offer benefits in improving physical fitness, mental health and social inclusion. It should, therefore, be a priority to comply with the UN Charter on the Rights of Persons with Disabilities and the 2030 Agenda for Sustainable Development Goals, in which attention to persons with disabilities, as well as other vulnerable groups, is a priority for the general improvement of society.

In the general population, it is recommended to raise the levels of physical activity in social isolation, adopting new strategies that promote physical activity in the current context, but, in people with visual impairment, it is a priority. Therefore, future research could address, among other aspects, the improvement of quality of life, physical condition and psychosocial health through physical activity and sport in all its manifestations.

## Figures and Tables

**Table 1:** Descriptive register of articles.

Study	Author/Year	Type	Group	Results	Citation
Motivational pathways to social and pedagogical inclusion in physical education	Wilhelmsen, Sørensen and Seippel (2019)	Interviews	64 children with disabilities	The progressive mastery of motor tasks makes the perception of social acceptance and inclusion increase in equal measure.	[21]
School inclusion: a study of cases of children with visual disabilities	Burgos, Rodelo and Jaramillo (2019)	Study of cases	20 children (19 without disabilities and 1 with visual impairment)	Awareness of people without disabilities and increased satisfaction and inclusion of people with disabilities through sports.	[22]
Physical activity of children and youth with disabilities and the effect on participation in meaningful leisure-time activities	Züll, Tillmann, Froböse and Anneken (2019)	Questionnaires and study of cases	152 females (37%), 256 males (63%)	Physical activity and shared sport have a positive effect on general health and inclusion.	[23]
Inclusion of students with visual disabilities in Physical Education classes.	Vila, Avendaño, Linzmayer, Mora, Duarte and Pacheco (2020)	Interviews and study of cases	12 people with visual disability	The training of teachers is a determining factor for inclusion; there is a serious problem of training in attention to disability in the classrooms.	[24]
Social inclusion and high school students with visual impairments	Jessop (2019)		12 people with visual disability	People feel more motivated and competent when they are in a situation of inclusion.	[25]
Association between education and health outcomes among adults with disabilities: evidence from Shanghai, China	Ge, Zhang, Lu, Chen, Sun and Li (2019)	Questionnaire	42,715 adults with disabilities	Physical activity encourages social inclusion.	[26]
Sports participation and quality of life in individuals with visual impairment	Ilhan, Idil and Ilhan (2021)	Questionnaire	200 adults with visual disability	Valuing quality of life.	[27]
La autoestima, la autonomía y el apoyo a lasnecesidades psicológicas básicas en personas con discapacidad visual	Mocha, Rosales, Chávez and Miranda (2019)	Intervention and questionnaire	40 adults with visual disability	Valuing autonomy and social inclusion.	[28]
The effect of sports on perceived quality of life of people with visual disability	Braga, Freitas, Dos Santosa, Oliveira, Pimentac and Ferraz (2018)	Questionnaire	37 adults with visual disability	Valuing quality of life.	[29]

**Table 2:** Quantitative map of the literature analysed.

Characteristics	Number (%)	Citations
PUBLICATIONS TYPE AND SOURCE		
Cross-sectional surveys	1 (11%)	[21]
Institutional case report	3 (33%)	[21,24,29]
Pilot feasibility study	5(64%)	[21,22,24,25,29]
Ecological study	1(11%)	[24]
Survey research with qualitative analysis	1(11%)	[20]
Quantitative analysis of contact with support services	6(66%)	[23,26,27,28,29]
CONDITIONS		
People with disabilities, overall	2 (22%)	[24,26]
People with visual disabilities	8(88%)	[21,22,23,25,26,27,28,29]
People with and without disabilities	1(11%)	[23]
Children/youth with disabilities (and their families)	2(22%)	[21,23]
Older adults experiencing disabilities	1(11%)	[25]

## Data Availability

Not applicable.
